# Clinical course and predictive risk factors for fatal outcome of SARS-CoV-2 infection in patients with chronic kidney disease

**DOI:** 10.1007/s15010-021-01597-7

**Published:** 2021-04-13

**Authors:** Lisa Pilgram, Lukas Eberwein, Kai Wille, Felix C. Koehler, Melanie Stecher, Siegbert Rieg, Jan T. Kielstein, Carolin E. M. Jakob, Maria Rüthrich, Volker Burst, Fabian Prasser, Stefan Borgmann, Roman-Ulrich Müller, Julia Lanznaster, Nora Isberner, Lukas Tometten, Sebastian Dolff, the LEOSS Study group, Lukas Tometten, Kai Wille, Siegbert Rieg, Stefan Borgmann, Matthias Wettstein, Nora Isberner, Maria Ruethrich, Christoph Spinner, Claudia Raichle, Mark Neufang, Frank Hanses, Bernd Hohenstein, Sven Stieglitz, Norma Jung, Robert Bals, Sebastian Dolff, Joerg Schubert, Maximilian Worm, Christian Degenhardt, Timo Brandenburger, Julia Fuerst, Maria Vehreschild, Ulrich Keller, Martin Hower, Michael von Bergwelt-Baildon, Jessica Rueddel, Katja de With, Beate Gruener, Lukas Eberwein, Beate Schultheis, David Heigener, Wolfgang Guggemos, Helga Peetz, Lorenz Walter, Juergen Prattes, Katja Rothfuss, Kerstin Hellwig, Jacob Nattermann, Uta Merle, Daniel Droehmann, Dominic Rauschning, Gabriele Mueller-Joerger, Alexander Weidemann, Christiane Piepel, Annika Ritter, Gernot Beutel, Janina Trauth, Anette Friedrichs, Wolfgang Bethge, Joerg Janne Vehreschild, Lisa Pilgram, Melanie Stecher, Maximilian Schons, Carolin E. M. Jakob, Annika Classen, Sandra Fuhrmann, Bernd Franke, Nick Schulze, Fabian Prasser, Martin Lablans

**Affiliations:** 1 Department of Internal Medicine, Hematology and Oncology, Goethe University Frankfurt, Frankfurt, Germany; 2 4th Department of Internal Medicine, Klinikum Leverkusen gGmbH, Leverkusen, Germany; 3 University Clinic for Haematology, Oncology, Haemostaseology and Palliative Care, Johannes Wesling Klinikum, University of Bochum, Minden, Germany; 4 Department II of Internal Medicine and Center for Molecular Medicine Cologne, Faculty of Medicine and University Hospital Cologne, University of Cologne, Cologne, Germany; 5 Faculty of Medicine and University Hospital Cologne, CECAD, University of Cologne, Cologne, Germany; 6 Department I of Internal Medicine, University Hospital of Cologne, University of Cologne, Cologne, Germany; 7 Division of Infectious Diseases, Department of Medicine II, Medical Centre - University of Freiburg, Faculty of Medicine, Freiburg, Germany; 8 Medical Clinic V, Academic Teaching Hospital Braunschweig, Brunswick, Germany; 9 Department of Internal Medicine II, University Hospital Jena, Jena, Germany; 10 Emergency Department, Faculty of Medicine and University Hospital Cologne, University of Cologne, Cologne, Germany; 11 Charite, University Hospital Berlin, Berlin Institute of Health (BIH), Anna-Louisa-Karsch-Str. 2, 10178 Berlin, Germany; 12 Charité – Universitätsmedizin Berlin, corporate member of Freie Universität Berlin, Humboldt-Universität Zu Berlin, and Berlin Institute of Health, Berlin, Germany; 13 Department of Infectious Diseases and Infection Control, Ingolstadt Hospital, Ingolstadt, Germany; 14 Systems Biology of Ageing Cologne (Sybacol), University of Cologne, Cologne, Germany; 15 Department of Internal Medicine 2, Klinikum Passau, Passau, Germany; 16 Division of Infectious Diseases, Department of Medicine II, University of Würzburg Medical Center, Würzburg, Germany; 17 Department of Gastroenterology and Infectiology, Klinikum Ernst-von-Bergmann, Potsdam, Germany; 18 Department of Infectious Diseases, West German Centre of Infectious Diseases, University Hospital Essen, University Duisburg-Essen, Hufelandstr. 55, 45122 Essen, Germany; 19 German Center for Infection Research (DZIF), Partner Site Bonn-Cologne, Cologne, Germany

**Keywords:** Chronic kidney disease, COVID-19, LEOSS, Predictive factor, SARS-CoV-2

## Abstract

**Purpose:**

The ongoing pandemic caused by the novel severe acute respiratory coronavirus 2 (SARS-CoV-2) has stressed health systems worldwide. Patients with chronic kidney disease (CKD) seem to be more prone to a severe course of coronavirus disease (COVID-19) due to comorbidities and an altered immune system. The study’s aim was to identify factors predicting mortality among SARS-CoV-2-infected patients with CKD.

**Methods:**

We analyzed 2817 SARS-CoV-2-infected patients enrolled in the Lean European Open Survey on SARS-CoV-2-infected patients and identified 426 patients with pre-existing CKD. Group comparisons were performed via Chi-squared test. Using univariate and multivariable logistic regression, predictive factors for mortality were identified.

**Results:**

Comparative analyses to patients without CKD revealed a higher mortality (140/426, 32.9% versus 354/2391, 14.8%). Higher age could be confirmed as a demographic predictor for mortality in CKD patients (> 85 years compared to 15–65 years, adjusted odds ratio (aOR) 6.49, 95% CI 1.27–33.20, *p* = 0.025). We further identified markedly elevated lactate dehydrogenase (> 2 × upper limit of normal, aOR 23.21, 95% CI 3.66–147.11, *p* < 0.001), thrombocytopenia (< 120,000/µl, aOR 11.66, 95% CI 2.49–54.70, *p* = 0.002), anemia (Hb < 10 g/dl, aOR 3.21, 95% CI 1.17–8.82, *p* = 0.024), and C-reactive protein (≥ 30 mg/l, aOR 3.44, 95% CI 1.13–10.45, *p* = 0.029) as predictors, while renal replacement therapy was not related to mortality (aOR 1.15, 95% CI 0.68–1.93, *p* = 0.611).

**Conclusion:**

The identified predictors include routinely measured and universally available parameters. Their assessment might facilitate risk stratification in this highly vulnerable cohort as early as at initial medical evaluation for SARS-CoV-2.

**Supplementary Information:**

The online version contains supplementary material available at 10.1007/s15010-021-01597-7.

## Introduction

In late 2019, SARS-CoV-2 broke out in China and subsequently expanded to a worldwide public health crisis with more several millions infected and more than 1 million deaths so far. COVID-19 is a respiratory syndrome characterized by fever, cough, and dyspnea with a broad clinical spectrum ranging from asymptomatic to fatal [1].

Kidney disease seems to be accompanied by worse outcome in COVID-19. SARS-CoV-2 interacts with the transmembrane protein angiotensin-converting enzyme 2 (ACE-2), best known for its role in the renin–angiotensin–aldosterone system (RAAS). ACE-2 is expressed in alveolar cells in the lung, as well as in the kidney, most abundant in proximal tubular cells and podocytes [2]. Pharmacological blockade of the RAAS increases cardiac and renal ACE-2 activity [3]. Remarkably, ACE-2 was first reported also as a functional viral receptor after the SARS epidemic in 2003 [4]. SARS-CoV-2 might cause direct tubular injury via direct viral toxicity which is supported by the detection of SARS-CoV-2 in human kidneys of autopsies by immunohistochemistry and in situ hybridization [5]. Kidney injury might also occur in the hyperinflammatory setting of COVID-19 due to hypoperfusion associated with resulting tubular injury and renal vasculitis, as well as by direct viral infection and replication in the kidney epithelial cells [6]. Volume depletion and concomitant use of nephrotoxic medications like nonsteroidal antiphlogistic drugs may worsen the decline of the glomerular filtration rate. In accordance with these considerations, acute kidney injury (AKI) has been identified as a relatively common finding among SARS-CoV-2-infected patients with severe clinical course and it is associated with respiratory failure and poor outcome [7–9].

Furthermore, patients with CKD seem to be prone to develop a more severe disease course of COVID-19 [10, 11]. There is a strong correlation between CKD and other comorbidities like hypertension, atherosclerotic cardiovascular diseases, and metabolic disturbances like obesity or insulin resistance which are all already identified risk factors for a severe clinical course of COVID-19. Moreover, uremia is associated with an impaired T cell response, causing an increased susceptibility to infections, viral cancers, and a reduced response to vaccinations. Additionally, some patients with CKD of autoimmune origin are treated with immunosuppressive medication. And lastly, patients with kidney failure in need of dialysis or a kidney transplant are older and frailer than other patient groups suffering from COVID-19 [12]. Patients undergoing hemodialysis may additionally have an increased risk of exposure to SARS-CoV-2 during their routine dialysis sessions, but might also benefit from intermittent anticoagulation during dialysis sessions.

However, there are only limited transregional and -sectoral data from European populations on COVID-19 in highly vulnerable CKD patients. We, therefore, analyzed polymerase chain reaction (PCR) or rapid test-proven SARS-CoV-2 cases from both in- and outpatient settings enrolled in Lean European Open Survey on SARS-CoV-2-infected patients (LEOSS) [13]. The main goal of this study was to investigate the clinical impact of COVID-19 in individuals with underlying CKD and to identify predictive factors for a fatal outcome of COVID-19 disease in this highly vulnerable cohort.

## Materials and methods

### Study design and patient cohort

This analysis was performed based on data from the transregional and transsectoral *LEOSS* registry. Patients with PCR- or rapid test-confirmed SARS-CoV-2 infection were included between March 16, 2020 and August 06, 2020 from 105 study sites. The dataset exclusively consists of cases with specified information on the status at the end of the acute treatment setting and a clear statement on the presence of CKD. Patients with pre-existing CKD of any stage were the focus of the analyses, while the referential population with negated CKD served as a reference for comparing frequency distributions.

### Clinical data, covariables and endpoint

**Fig. 1:**
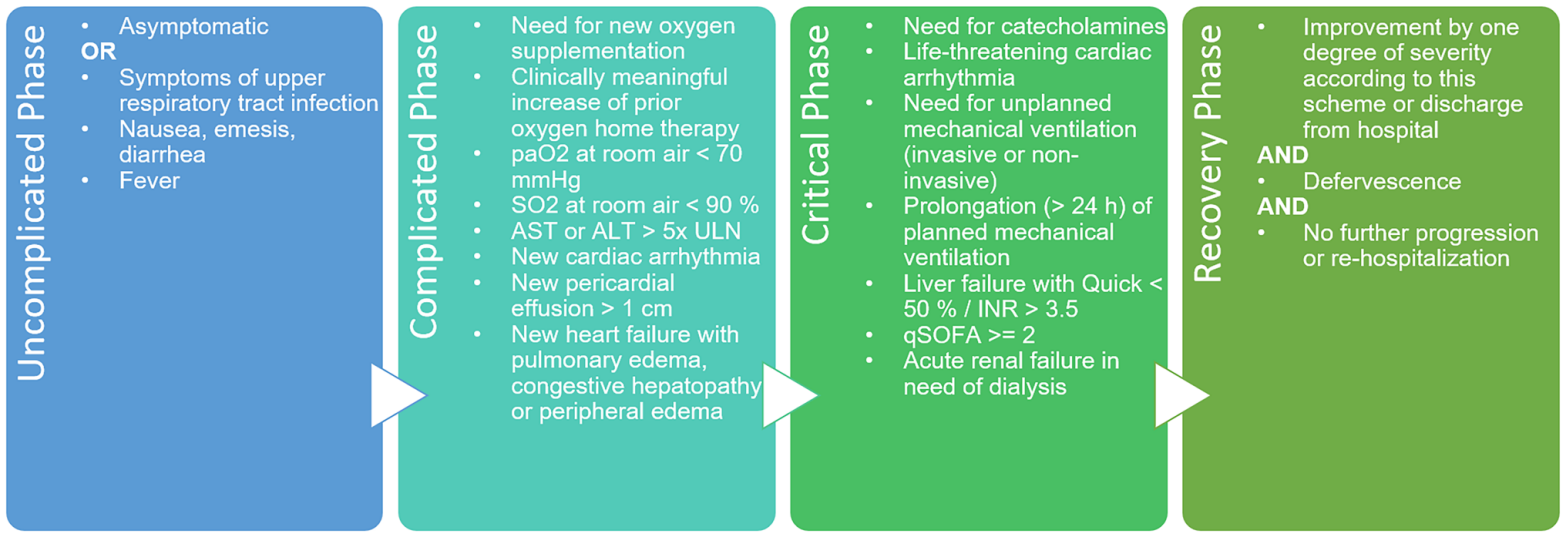
LEOSS definition of clinical phases (https://leoss.net/statistics/).* ALT* alanine transaminase, *AST* aspartate aminotransferase, *INR* international normalized ratio, *SO*_*2*_ oxygen saturation, *ULN* upper limit of normal in the respective local laboratory

Clinical data were reported in an electronic case report form using the online cohort platform ClinicalSurveys.net. ClinicalSurveys.net was developed by the University Hospital of Cologne (UHC) and is hosted by QuestBack, Oslo, Norway on servers of UHC, Cologne, Germany, as part of a software-as-a-service agreement. Data were processed on the servers of UHC. Anonymous patient enrollment into the LEOSS registry was performed retrospectively at the end of the acute treatment setting. To prevent re-identification, data were aggregated over time in uncomplicated, complicated, critical and recovery phase which were defined by clinical and/or laboratory findings (see Fig. 1). Additional information about data acquisition in LEOSS can be found under https://leoss.net.

The diagnostic parameters of this analysis were determined closest to the first positive SARS-CoV-2 testing but did not exceed 48 h after testing. Continuous parameters were vertically aggregated into categories. Age categories ≤ 65 years were summarized into one category due to the low number of patients with underlying CKD in the respective categories. Further patients’ characteristics such as sex, BMI, comorbidities, smoking status and medication (ACE inhibitors or ARBs, immunosuppressive medication) were included in the regression models. Information regarding country of residence, details of the pre-existing CKD and the clinical course were used descriptively. The pre-existing condition was documented by investigators according to anamnestic diagnosis and according to KDIGO guidelines as well as diagnoses during the course of disease (e.g., acute kidney injury, AKI). Diagnostic factors of primary interest were parameters assessed in routine basic assessment evaluated via missing rate, health economic aspects and clinical expertise. Vital signs and clinical findings (body temperature, oxygen saturation, dyspnea) as well as laboratory values (LDH, leukocytes, lymphocytes, platelets, hemoglobin, CRP) were chosen as covariables in the regression models. Death within the observational period was used as end-point for this analysis.

### Statistical analysis

We described patients’ characteristics as absolute numbers and percentages, continuous measures as medians and IQRs. Group comparisons to patients without pre-existing CKD were carried out using the *χ*^2^ test or the Mann–Whitney *U* test. To control the problem of multiple comparisons, the Bonferroni correction was used. Predictive factors (covariables) for mortality (dependent variable) were identified via univariate and multivariable logistic regression models. Covariables for multivariable regression were chosen according to their significance below the 0.1 significance level in univariate modeling and added via enter method, further adjustments were evaluated using Akaike information criterion (AIC) and Bayesian information criterion (BIC). Multicollinearity problems were identified using the variance inflation factor (VIF). The strength of association was assessed using odds ratios (ORs) with 95% confidence intervals (CIs). The level of significance was chosen to be *p* < 0.05. Sensitivity analyses by transferring the final model to a sub-cohort without the need for dialysis, and by conducting the model with more restrictive selection of covariables were used to confirm robustness of the model. Missing rates were obtained and further analyzed for data with a missing rate of > 5%. Missing mechanisms were addressed using graphical (correlation heat map) and statistical methods (frequency distribution, group differences) to exclude an association with the endpoint before being excluded from the analysis.

All data management and statistical analysis were conducted using Python (Python Software Foundation, version 3.7.6.) on Jupyter Notebook (Available at https://jupyter.org/).

### Ethical statement

Data were recorded anonymous without any patient-identifying data. Data were categorized and aggregated over time. To prevent re-identification, further anonymization steps were taken (see https://leoss.net for more information). LEOSS was approved by the applicable local ethics committees of all participating centers and registered at the German Clinical Trials Register (DRKS, No. S00021145).

## Results

### Cohort and patient characteristics

A total of 2817 SARS-CoV-2-infected patients from 105 registered study sites were enrolled in *LEOSS* between March 16, 2020 and August 06, 2020 and considered valid for analysis. We identified 426/2817 (15.2%) patients with pre-existing CKD. 2391/2817 (84.9%) SARS-CoV-2-infected patients without underlying CKD were considered as referential population. CKD patients’ characteristics are depicted in detail in Table 1 (excluding the respective missing values). The majority of CKD patients was aged 76 and older (257/426, 60.3%), 175/426 (41.1%) females and 419/426 (98.4%) living in Germany. Apart from CKD, most patients were suffering from at least one more comorbidity (hypertension 339/420, 80.7%; chronic heart failure 132/399, 33.1%; atrial fibrillation 135/416, 32.5%; coronary heart disease 133/397, 33.5%; cerebrovascular disease 76/408, 18.6%; diabetes mellitus 171/416, 41.1%; chronic obstructive pulmonary disease (COPD) 53/416, 12.7%; oncological disease 85/411, 20.7%), and showed an elevated body mass index (BMI) (≥ 25 kg/m^2^ 167/292, 57.2%).

**Table 1:** Characteristics of SARS-COV-2-infected patients suffering from chronic kidney disease

	Study cohort: Chronic kidney disease
Included cases	426
Age—no. (%)	
15–65	86 (20.2)
66–75	83 (19.5)
76–85	169 (39.7)
> 85	88 (20.7)
Sex—no. (%)	
Female	175 (41.1)
Male	251 (58.9)
Country of residence—no. (%)	
Germany	419 (98.4)
Turkey	3 (0.7)
Austria	3 (0.7)
Spain	1 (0.2)
BMI—no. (%)	
< 18.5 kg/m^2^	6 (2.1)
18.5–24.9 kg/m^2^	119 (40.8)
25–29.9 kg/m^2^	99 (33.9)
30–34.9 kg/m^2^	46 (15.7)
≥ 35 kg/m^2^	22 (7.5)
Comorbidities—no. (%)	
Hypertension	339 (80.7)
Chronic heart failure	132 (33.1)
Atrial fibrillation	135 (32.5)
Coronary heart disease	133 (33.5)
Cerebrovascular disease	76 (18.6)
Diabetes mellitus	171 (41.1)
COPD	53 (12.7)
Oncological disease^a^	85 (20.7)
GFR categories (KDIGO)—no. (%)	
G1 (GFR ≥ 90 ml/min)	17 (5.4)
G2 (GFR 60–89 ml/min)	42 (13.4)
G3 (GFR 30–59 ml/min)	149 (47.6)
G4 (GFR 15–29 ml/min)	41 (13.1)
G5 (GFR < 15 ml/min)	64 (20.5)
Causes of CKD—no. (%)	
Vascular-hypertensive disease	70 (50.4)
Secondary glomerular disease	30 (21.6)
Primary glomerular disease	13 (9.3)
Idiopathic kidney disease	10 (7.2)
Obstructive nephropathy	7 (5.0)
Tubulointerstitial disease	4 (2.9)
Polycystic kidney disease	4 (2.9)
Congenital disease	1 (0.7)
Kidney transplantation—no. (%)	
Kidney transplantation	25 (6.1)
Dialysis—no. (%)	
On dialysis	75 (18.2)
Hemodialysis	62 (96.9)
Peritoneal dialysis	2 (3.1)
Smoking status—no. (%)	
Active smoker	27 (13.2)
Former smoker	41 (20.0)
Non smoker	137 (66.8)
Medication—no. (%)	
ACE inhibitors or ARBs^b^	222 (55.2)
Immunosuppressive medication^c^	74 (19.7)

Continuous parameters were collected in categories. All variables are expressed as numbers (no.) and percentages (%) referred to the numbers excluding missing data. Missing rates and frequency distribution are displayed in Suppl. Table 2 for variables with missing rate > 5%

*BMI* body mass index, *COPD* chronic obstructive pulmonary disease, *GFR* glomerular filtration rate, *CKD* chronic kidney disease, *KDIGO* Kidney Disease: Improving Global Outcomes, *ACE* *inhibitors* angiotensin-converting enzyme inhibitor, *ARBs* angiotensin II receptor blocker

^a^Leukemia, lymphoma or solid tumor

^b^At first positive SARS-CoV-2 detection

^c^Within the last 3 months

Almost half of the CKD patients (149/313, 47.6%) were classified as stage G3 (GFR 30–59 ml/min) according to Kidney Disease Improving Global Outcome (KDIGO) classification. The most common reported cause of CKD was vascular-hypertensive disease (70/139, 50.4%) followed by secondary glomerular disease (30/139, 21.6%). A history of kidney transplantation was documented in 25/407 (6.1%) patients, 75/412 (18.2%) patients were on dialysis, predominantly on hemodialysis (62/64, 96.9%).

Immunosuppressive treatment was frequent in CKD patients due to different reasons. 74/375 (19.7%) patients with underlying CKD received immunosuppressive medication. A history of kidney transplantation was a main reason and specified in 25/68 (36.8%) patients; in 43/68 (63.2%), immunosuppressive medication was indicated for other reasons. The following comorbidities were present in this sub-cohort (excluding the respective missing values): hypertension 55/74 (74.3%), chronic heart failure 16/72 (22.2%), atrial fibrillation 15/73 (20.5%), coronary heart disease 14/72 (19.4%), cerebrovascular disease 6/71 (8.5%), diabetes mellitus 29/74 (39.2%), COPD 5/73 (6.9%), and oncological disease 24/72 (33.3%).

### Clinical course of SARS-CoV-2 infection in patients with underlying CKD

Patients were admitted to hospital in 97.1%, the median inpatient stay was 13 days [interquartile range (IQR) 7–21 days]. At first positive SARS-CoV-2 testing, symptoms were present in 293/339 (86.4%) of the study cohort; among those, respiratory symptoms such as rhinorrhea, sore throat, dry or productive cough were present in 107/268 (32.6%), dyspnea, respectively, in 86/278 (25.5%). Less frequently patients suffered from gastrointestinal symptoms (44/282, 15.6%), weakness (64/280, 22.9%) and smell or taste disorder (5/335, 1.5%). At the time of the first positive SARS-CoV-2 testing, 229/423 (54.1%) of the patients were classified as uncomplicated according to the LEOSS phases (Fig. 1). From our 426 CKD patients, 56/342 (16.4%,) have additionally suffered from AKI at baseline. In the course of disease, 112/300 (37.3%) of the patients were admitted to the intensive care unit (ICU) and 140/426 (32.9%) died in the course of disease, although only 67/413 (16.2%) needed invasive ventilation (see Table 2).

**Table 2:** Clinical course of SARS-CoV-2 infection in patients suffering from chronic kidney disease

	Study cohort: Chronic kidney disease
Symptoms at first positive SARS-CoV-2 detection—no. (%)	
Symptomatic	293 (86.4)
Dyspnea	86 (25.5)
Other respiratory symptoms^a^	107 (32.6)
Gastrointestinal symptoms^b^	44 (12.7)
Weakness^c^	64 (18.7)
Smell or taste disorder	6 (1.8)
Phase^d^ at first positive SARS-CoV-2 detection—no. (%)	
Uncomplicated phase	229 (54.1)
Complicated phase	163 (38.5)
Critical phase	19 (4.5)
Recovery phase	0 (0.0)
Dead	12 (2.8)
Phases^d^ in the course of disease—no. (%)	
Uncomplicated phase	333 (78.2)
Complicated phase	273 (64.1)
Critical phase	118 (27.7)
Hospitalization^e^ in the course of disease—no. (%)	
Inpatient treatment	304 (97.1)
ICU treatment	112 (37.3)
Length of inpatient treatment^e^—median (IQR) days	
Duration of overall inpatient stay	13 (7–21)
Duration of ICU stay	11 (5–19)
Outcomes—no. (%)	
Invasive ventilation	67 (16.2)
Death	140 (32.9)

Continuous variables are expressed as median and interquartile range (IQR), categorical variables as numbers (no.) and percentages (%) referred to the numbers excluding missing data. Missing rates and frequency distribution are displayed in Suppl. Table 2 for variables with missing rate > 5%

*ICU* intensive care unit

^a^Other respiratory symptoms include runny nose, sore throat, dry or productive cough

^b^Gastrointestinal symptoms include diarrhea, nausea or emesis

^c^Weakness includes muscle weakness or excessive tiredness

^d^Phases according to the LEOSS definition as shown in Fig. 1

^e^Patients receiving inpatient and/or ICU treatment for more than 4 days before the detection of SARS-CoV-2 were not considered

### Comparative descriptive analysis

Compared to the referential population from the *LEOSS* registry, frequency distributions differed regarding age (≥ 76 years 592/2391, 24.8%, *p* < 0.001; numbers indicated for the reference population, see abstract above for CKD) and comorbidities (hypertension 1043/2376, 43.9%, *p* < 0.001; chronic heart failure 150/2362, 6.4%, *p* < 0.001, atrial fibrillation 239/2366, 10.1%, *p* < 0.001; coronary heart disease 307/2344, 13.1%, *p* < 0.001; cerebrovascular disease 182/2372, 7.7%, *p* < 0.001; diabetes mellitus 391/2383, 16.4%, *p* < 0.001; COPD 112/2385, 4.7%, *p* < 0.001; oncological disease 315/2377, 13.3%, *p* = 0.003). In comparison to CKD patients, the proportion of AKI at baseline was lower 86/1839 (4.7%, *p* < 0.001). There were significant differences in mortality (354/2391, 14.8%, *p* < 0.001) between these groups while showing comparable prevalence of ICU admission (623/1866, 33.4%, *p* = 0.773) and invasive ventilation (417/2331, 17.9%, *p* = 0.955).

### Baseline predictive factors for mortality in patients with pre-existing CKD

We analyzed patient characteristics and basic diagnostic assessment at first positive SARS-CoV-2 testing to identify baseline factors predicting mortality of COVID-19 in patients with pre-existing CKD (Table 3). Adjusted risk factors included higher age (> 85 years compared to the age 15–65 years, aOR 6.49, 95% CI 1.27–33.20, *p* = 0.025), markedly elevated lactate dehydrogenase (LDH) (> 2 × upper limit of normal (ULN) compared to the reference range, aOR 23.21, 95% CI 3.66–147.11, *p* < 0.001), thrombocytopenia (< 120,000/µl, aOR 11.66, 95% CI 2.49–54.70, *p* = 0.002), anemia (Hb < 10 g/dl, aOR 3.21, 95% CI 1.17–8.82, *p* = 0.024) and strongly elevated c-reactive protein (CRP) (≥ 30 mg/l, aOR 3.44, 95% CI 1.13–10.45, *p* = 0.029). However, pre-existing comorbidities and dialysis seem not to be relevant prognostic factors in CKD patients. When adjustment for covariables was limited to parameters identified as predictive via univariate modeling on a significance level of 0.05, except for CRP (≥ 30 mg/l, aOR 2.09, 95% CI 0.93–4.70, *p* = 0.074), predictive factors remained robust (supplementary Table 3).

**Table 3:** Frequency distribution, univariate and multivariable logistic regression of predictive factors for mortality in SARS-CoV-2-infected patients suffering from chronic kidney disease

	Frequency distribution			Univariate model			Mutivariable model		
	Mortality	Alive	*p *value	OR	95% CI	*p *value	aOR	95% CI	*p *value
Included cases—no. (%)	140 (32.9)	286 (67.1)							
Age—no. (%)									
15–65	14 (10.0)	72 (25.2)	0.002	Reference	Reference	Reference	Reference	Reference	Reference
66–75	21 (15.0)	62 (21.7)		1.74	0.82–3.71	0.151	0.85	0.15–4.97	0.860
76–85	62 (44.3)	107 (37.4)		2.98	1.55–5.72	0.001	1.65	0.36–7.63	0.520
> 85	43 (30.7)	45 (15.7)		4.91	2.42–9.98	< 0.001	6.49	1.27–33.20	0.025
Sex—no. (%)									
Female	52 (37.1)	123 (43.0)	0.855	Reference	Reference	Reference	*	*	*
Male	88 (62.9)	163 (57.0)		1.28	0.84–1.93	0.248	*	*	*
BMI—no. (%)									
< 18.5 kg/m^2^	3 (3.5)	3 (1.5)	0.996	2.84	0.54–14.81	0.216	*	*	*
18.5–24.9 kg/m^2^	31 (36.5)	88 (42.5)		Reference	Reference	Reference	*	*	*
25.0–29.9 kg/m^2^	30 (35.3)	69 (33.3)		1.23	0.68–2.23	0.486	*	*	*
30.0–34.9 kg/m^2^	14 (16.5)	32 (15.5)		1.24	0.59–2.63	0.571	*	*	*
≥ 35.0 kg/m^2^	7 (8.2)	15 (7.3)		1.32	0.49–3.55	0.576	*	*	*
Comorbidities—no. (%)^a^									
Hypertension	116 (84.7)	223 (78.8)	0.727	1.49	0.86–2.56	0.154	*	*	*
Chronic heart failure	55 (41.4)	77 (29.0)	0.187	1.73	1.12–2.67	0.013	1.14	0.39–3.33	0.813
Atrial fibrillation	55 (39.6)	80 (28.9)	0.306	1.61	1.05–2.47	0.029	0.72	0.24–2.19	0.566
Coronary heart disease	50 (38.8)	83 (31.0)	0.668	1.41	0.91–2.19	0.124	*	*	*
Cerebrovascular disease	32 (23.7)	44 (16.1)	0.489	1.62	0.97–2.70	0.065	1.65	0.55–4.93	0.368
Diabetes mellitus	61 (43.9)	110 (39.7)	0.955	1.19	0.79–1.79	0.415	*	*	*
COPD	19 (13.8)	34 (12.2)	0.995	1.15	0.63–2.09	0.658	*	*	*
Oncological disease^b^	25 (18.3)	60 (21.9)	0.946	0.80	0.47–1.34	0.900	*	*	*
Dialysis—no. (%)^a^									
On dialysis	27 (19.6)	48 (17.5)	0.992	1.15	0.68–1.93	0.611	1.17	0.28–4.91	0.826
Smoking status—no. (%)									
Active smoker	8 (11.4)	19 (14.1)	0.998	0.76	0.31–1.85	0.541	*	*	*
Former smoker	13 (18.6)	28 (20.7)		0.83	0.40–1.76	0.632	*	*	*
Non smoker	49 (70.0)	88 (65.2)		Reference	Reference	Reference	*	*	*
Medication—no. (%)^a^									
ACE inhibitors or ARBs^c^	64 (49.6)	158 (57.9)	0.659	0.72	0.47–1.09	0.120	*	*	*
Immunosuppressive medication^d^	17 (14.2)	57 (22.4)	0.485	0.57	0.32–1.04	0.065	0.76	0.20–2.89	0.691
Vital signs^c^—no. (%)^a^									
Body temperature ≥ 38 °C	41 (36.9)	69 (31.7)	0.921	1.26	0.78–2.04	0.337	*	*	*
SO_2_ < 90%	32 (29.4)	44 (20.9)	0.580	1.58	0.93–2.68	0.092	0.44	0.14–1.41	0.167
Dyspnea	39 (35.1)	47 (20.8)	0.090	2.06	1.24–3.42	0.004	2.72	0.95–7.80	0.063
LDH^c^—no. (%)									
Normal	20 (20.8)	65 (35.5)	0.009	Reference	Reference	Reference	Reference	Reference	Reference
ULN–2 × ULN	55 (57.3)	106 (57.9)		1.69	0.93–3.07	0.087	2.09	0.67– 6.58	0.206
> 2 × ULN	21 (21.9)	12 (6.6)		5.69	2.39–13.55	< 0.001	23.21	3.66–147.11	< 0.001
Leukocytes^c^—no. (%)									
< 4000/µl	19 (15.8)	39 (17.8)	0.958	0.92	0.50–1.68	0.780	*	*	*
4000–11,999/µl	85 (70.8)	160 (73.1)		Reference	Reference	Reference	*	*	*
> 12,000/µl	16 (13.3)	20 (9.1)		1.51	0.74–3.06	0.257	*	*	*
Lymphocytes^c^—no. (%)^a^									
< 800/µl	55 (62.5)	77 (44.0)	0.091	2.12	1.26–3.58	0.005	0.47	0.16–1.36	0.163
Platelets^c^—no. (%)^a^									
< 120,000/µl	27 (22.7)	20 (9.3)	0.022	2.88	1.53–5.40	< 0.001	11.66	2.49–54.70	0.002
Hemoglobin^c^—no. (%)^a^									
< 10 g/dl	46 (38.0)	56 (25.8)	0.240	1.76	1.09–2.84	0.020	3.21	1.17–8.82	0.024
CRP^c^—no. (%)^a^									
≥ 30 mg/l	87 (73.7)	115 (53.0)	0.008	2.49	1.53–4.06	< 0.001	3.44	1.13–10.45	0.029

Continuous parameters were collected in categories. All variables are expressed as numbers (no.) and percentages (%) referred to the numbers excluding missing data. Missing rates and frequency distribution are displayed in Suppl. Table 2 for variables with missing rate > 5%. Variance inflating factors are demonstrated in Suppl. Table 1. *n* = 289 observations were excluded from multivariable regression model due to missingness

*OR* odds ratio, *aOR* adjusted odds ratio, *CI* confidence interval, *BMI* body mass index, *COPD* chronic obstructive pulmonary disease, *ACE inhibitors* angiotensin-converting enzyme inhibitor, *ARBs* angiotensin II receptor blocker, *SO*_*2*_ oxygen saturation in arterial blood, *LDH* lactate dehydrogenase, *ULN* upper limit of normal in the respective local laboratory, *CRP* C-reactive protein

^*^Excluded due to model quality

^a^No reference level indicated in binary variables

^b^Leukemia, lymphoma or solid tumor

^c^At first positive SARS-CoV-2 detection

^d^Within the last 3 months

When limiting the regression modeling to patients who are affected by CKD but not requiring dialysis, these prior-described baseline prognostic factors except for anemia could be confirmed (Table 4). Markedly elevated LDH was once again identified as the strongest predictor for mortality (> 2 × ULN compared to the reference range, aOR 34.35, 95% CI 3.98–296.21, *p* = 0.001). Positive hemoglobin or erythrocytes in urine test strips showed a tendency to predict mortality in univariate modeling not being significant on the 0.05 significance level (OR 2.08, 95% CI 1.00–4.33, *p* = 0.050). Further additional parameters, such as creatinine and further urine test strips parameters (leukocytes, protein) that were easily assessable in this CKD sub-cohort, did not show a significant effect on the outcome in univariate modeling either.

**Table 4:** Frequency distribution, univariate and multivariable logistic regression of predictive factors for mortality in SARS-CoV-2-infected patients suffering from chronic kidney disease not on dialysis

	Frequency distribution			Univariate model			Mutivariable model		
	Mortality	Alive	*p* value	OR	95% CI	*p* value	aOR	95% CI	*p* value
Included cases—no. (%)	111 (32.9)	226 (67.1)							
Age—no. (%)									
15–65	9 (8.1)	53 (23.5)	0.001	Reference	Reference	Reference	Reference	Reference	Reference
66–75	13 (11.7)	50 (22.1)		1.53	0.60–3.89	0.371	1.83	0.24–14.01	0.560
76–85	52 (46.9)	87 (38.5)		3.52	1.60–7.72	0.002	2.71	0.42–17.38	0.294
> 85	37 (33.3)	36 (15.9)		6.05	2.61–14.06	< 0.001	14.01	1.98–99.40	0.008
Sex—no. (%)									
Female	40 (36.0)	98 (43.4)	0.799	Reference	Reference	Reference	*	*	*
Male	71 (64.0)	128 (56.6)		1.36	0.85–2.17	0.199	*	*	*
BMI—no. (%)									
< 18.5 kg/m^2^	2 (3.3)	2 (1.3)	0.937	3.68	0.49–27.90	0.207	*	*	*
18.5–24.9 kg/m^2^	19 (31.2)	70 (44.6)		Reference	Reference	Reference	*	*	*
25.0–29.9 kg/m^2^	24 (39.3)	47 (29.9)		1.88	0.93–3.81	0.079	*	*	*
30.0–34.9 kg/m^2^	11 (18.0)	25 (15.9)		1.62	0.68–3.07	0.277	*	*	*
≥ 35.0 kg/m^2^	5 (8.2)	13 (8.3)		1.42	0.45–4.47	0.552	*	*	*
Comorbidities—no. (%)^a^									
Hypertension	95 (88.0)	177 (79.0)	0.414	1.94	1.00–3.77	0.050	*	*	*
Chronic heart failure	44 (41.1)	60 (28.2)	0.244	1.78	1.09–2.90	0.020	1.31	0.42–4.04	0.643
Atrial fibrillation	41 (36.9)	63 (28.1)	0.611	1.50	0.92–2.43	0.102	0.60	0.19–1.95	0.400
Coronary heart disease	37 (36.6)	63 (29.3)	0.789	1.39	0.85–2.30	0.192	*	*	*
Cerebrovascular disease	25 (23.6)	31 (13.9)	0.312	1.91	1.06–3.44	0.031	2.07	0.61–7.07	0.245
Diabetes mellitus	49 (44.1)	87 (38.5)	0.912	1.26	0.80–2.00	0.321	*	*	*
COPD	15 (13.6)	23 (10.3)	0.934	1.38	0.69–2.76	0.364	*	*	*
Oncological disease^b^	22 (20.2)	56 (24.9)	0.923	0.76	0.44–1.33	0.341	*	*	*
Smoking status—no. (%)									
Active smoker	5 (8.6)	14 (13.0)	0.970	0.59	0.20–1.75	0.338	*	*	*
Former smoker	11 (19.0)	25 (23.2)		0.72	0.32–1.62	0.430	*	*	*
Non smoker	42 (72.4)	69 (63.8)		Reference	Reference	Reference	Reference	Reference	Reference
Medication—no. (%)^a^									
ACE inhibitors or ARBs^c^	55 (52.9)	125 (57.3)	0.967	0.84	0.52–1.34	0.450	*	*	*
Immunosuppressive medication^d^	12 (12.2)	47 (23.4)	0.271	0.46	0.23–0.91	0.025	0.69	0.15–3.16	0.628
Vital signs^c^—no. (%)^a^									
Body temperature ≥ 38 °C	30 (34.9)	50 (27.6)	0.833	1.40	0.81–2.43	0.227	*	*	*
SO_2_ < 90%	61 (30.7)	147 (18.3)	0.269	1.97	1.09–3.56	0.024	0.51	0.15–1.76	0.290
Dyspnea	29 (33.7)	38 (20.7)	0.252	1.95	1.10–3.46	0.022	2.25	0.68–7.41	0.182
LDH^c^—no. (%)									
Normal	10 (13.5)	54 (35.1)	< 0.001	Reference	Reference	Reference	Reference	Reference	Reference
ULN–2 × ULN	46 (62.2)	91 (59.1)		2.73	1.27–5.85	0.010	3.70	0.87—15.72	0.077
> 2 × ULN	18 (24.3)	9 (5.8)		10.80	3.79–30.76	< 0.001	34.35	3.98–296.21	0.001
Leukocytes^c^—no. (%)									
< 4000/µl	13 (13.7)	31 (16.7)	0.985	0.83	0.41–1.68	0.599	*	*	*
4000–11,999/µl	69 (72.6)	136 (73.1)		Reference	Reference	Reference	*	*	*
> 12,000/µl	13 (13.7)	19 (10.2)		1.34	0.63–2.89	0.442	*	*	*
Lymphocytes^c^—no. (%)^a^									
< 800/µl	28 (60.6)	90 (40.8)	0.108	2.23	1.25–3.96	0.006	0.59	0.19–1.80	0.355
Platelets^c^—no. (%)^a^									
< 120,000 /µl	23 (24.5)	16 (8.7)	0.011	3.42	1.71–6.86	< 0.001	12.10	2.06–70.97	0.006
Hemoglobin^c^—no. (%)^a^									
< 10 g/dl	32 (33.3)	45 (24.3)	0.631	1.56	0.91–2.67	0.110	1.95	0.64–5.94	0.240
CRP^c^—no. (%)^a^									
CRP ≥ 30 mg/l	69 (74.2)	97 (52.7)	0.018	2.58	1.49–4.46	< 0.001	3.60	1.06–12.24	0.040
Creatinine^c^—no. (%)^e^									
Normal	23 (24.0)	53 (28.8)	0.749	Reference	Reference	Reference	*	*	*
ULN–2 × ULN	53 (55.2)	95 (51.6)		1.29	0.71–2.33	0.407	*	*	*
> 2 × ULN	20 (20.8)	36 (19.6)		1.28	0.61–2.67	0.509	*	*	*
Urine test strip^c^—no. (%)^a,e^									
Leukocytes positive	21 (44.7)	33 (37.5)	0.956	1.34	0.66–2.76	0.418	*	*	*
Protein positive	29 (69.1)	48 (55.2)	0.687	1.81	0.83–3.95	0.134	*	*	*
Hemoglobin positive	30 (63.8)	39 (45.9)	0.419	2.08	1.00–4.33	0.050	*	*	*

Continuous parameters were collected in categories. All variables are expressed as numbers (no.) and percentages (%) referred to the numbers excluding missing data. Missing rates and frequency distribution are displayed in Suppl. Table 2 for variables with missing rate > 5%. *n* = 219 observations were excluded from multivariable regression model due to missingness

*OR* odds ratio, *aOR* adjusted odds ratio, *CI* confidence interval, *BMI* body mass index, *COPD* chronic obstructive pulmonary disease, *ACE inhibitors* angiotensin-converting enzyme inhibitor, *ARBs* angiotensin II receptor blocker, *SO*_*2*_ oxygen saturation in arterial blood, *LDH* lactate dehydrogenase, *ULN* upper limit of normal in the respective local laboratory, *CRP* C-reactive protein

^*^Parameters chosen as for whole population including patients on dialysis

^a^No reference level indicated in binary variables

^b^Leukemia, lymphoma or solid tumor

^c^At first positive SARS-CoV-2 detection

^d^Within the last 3 months

^e^Parameters only included in patients without dialysis due to unclear interpretation in dialysis patients.

Additional nonstandard laboratory parameters exhibited high missing rates > 50% in basic assessment (see supplementary Table 2). Among those, elevated procalcitonin (> 0.5 ng/ml, OR 2.91, 95% CI 1.54–5.49, *p* < 0.001), interleukin 6 (≥ 50 pg/ml, OR 5.54, 95% CI 1.69–18.18, *p* = 0.005) and troponin T (> 2 × ULN, OR 8.45, 95% CI 2.57–27.74, *p* < 0.001) were associated with mortality in univariate modeling (supplementary Table 4).

## Discussion

The present study analyzed clinical characteristics and outcomes of SARS-CoV-2-infected patients with emphasis on CKD obtained from the *LEOSS* registry, a European multi-center cohort study of SARS-CoV-2-infected patients from 105 registered sites. A high prevalence of CKD was found in infected patients in this registy. However, at 15.2%, the overall proportion of patients with CKD is similar to other recently published reports, and it reflects the percentage of patients with CKD in the general population, that reached almost 15% in the US in 2017 [14]. A strength of the current analysis is the description of characteristics of hospitalized and outpatient patients including demographics, comorbidities, outcomes and current treatments in a large sample size derived from transsectoral health care facilities in various but predominantly European countries.

Previous studies revealed various risk factors for SARS-CoV-2-infected patients including CKD. The current study demonstrates high mortality of more than 30% in our CKD cohort, which is twice as high as in our reference population. Remarkably, admission to ICU and the need for invasive ventilation did not significantly differ from the referential population. These findings suggest that CKD patients are not particularly at risk of invasive ventilation which is in contrast to the association between AKI and invasive ventilation. Considering the detected higher mortality rates, this observation could be due to organ failure apart from respiratory insufficiency but also might be a consequence of accomplished patient decrees in the context of the high rate of severe comorbidities and the older age in the CKD population.

In accordance with multiple previous studies, higher age was associated with a worse outcome [1, 15, 16]. Older age is also associated with a higher risk of hypertension which is a described prognostic factor in COVID-19 [17]. Noteworthy, hypertension is, as with our patients, generally a very common clinical finding in CKD patients. However, we did not find an association between hypertension neither for other cardiovascular diseases and adverse outcome in our cohort. Patients with CKD are often treated with ACE inhibitors or angiotensin receptor blockers (ARBs), which could increase the expression of ACE-2 in these patients. The use as pre-medication seems not to affect the outcome which is in accordance with already published data [18, 19].

End-stage kidney disease (ESKD) and associated kidney replacement therapy (KRT) were considered to be another risk factor predicting adverse outcome. A retrospective study from New York identified higher in-hospital mortality in ESKD 20, 21. An observational study from Germany identified the need for dialysis as a risk factor in patients receiving mechanical ventilation, but without being able to distinguish between patients on preexisting KRT and AKI [14]. In contrast, the French REIN Registry did not find increased mortality in chronic dialysis patients [21]. The present data demonstrate that mortality in CKD patients is independent of kidney replacement therapy. The REIN Registry and our data from *LEOSS* offer a more transsectoral point of view resulting in the inclusion of more patients from areas with less stressed health care systems and probably of less severely ill patients which might be a possible explanation of the heterogeneous results. A limitation of the present study is the unprecise cause of CKD which is related to the study design. All patients’ characteristics were at admission which provides a homogeneous data set. The drawback is that many emergency departments and hospitals have difficulties to assess proteinuria apart from dip stick analysis to quantify albuminuria. In our CKD cohort, 38.1% of analyzed urine samples had an albuminuria CKD grade A2 (34.0% A1, 27.8% A3). However, the proportion of lacking albuminuria is too high to draw conclusions. Ideally, also microscopic sediments were performed but in routine diagnostic this is also no standard procedure.

We identified anemia, thrombocytopenia, strongly elevated levels of LDH (> 2 × ULN) and CRP (> 30 mg/dl) at initial presentation as factors predicting a severe course of COVID-19 in patients with pre-existing renal impairment. Lymphopenia and elevated levels of LDH have been reported in several studies as significant findings in patients with pneumonia due to SARS-CoV-2 [1, 22–24]. Anemia and thrombocytopenia have not been identified as major predictive factors in these cohorts but have been described as such in other contexts [25, 26]. Especially, the presence of thrombocytopenia seems to be associated with poor outcome in hospitalized patients with COVID-19. [27] COVID-19-associated thrombocytopathy due to a pathological platelet hyperactivation serves as an explanation of this observation, possibly induced by cardiovascular vascular risk factors like old age, diabetes mellitus, obesity and conditions with increased levels of reactive oxygen species—all of them are common in CKD patients and might also contribute to the findings of our study [28].

These differences might either be explained by a strong dependence on the specific patient cohort, by divergent threshold values, or by the combination of both. Further routine parameters of baseline assessment in the context of COVID-19 such as body temperature, oxygen saturation or dyspnea do not serve as adequate predictive parameters.

**Fig. 2:**
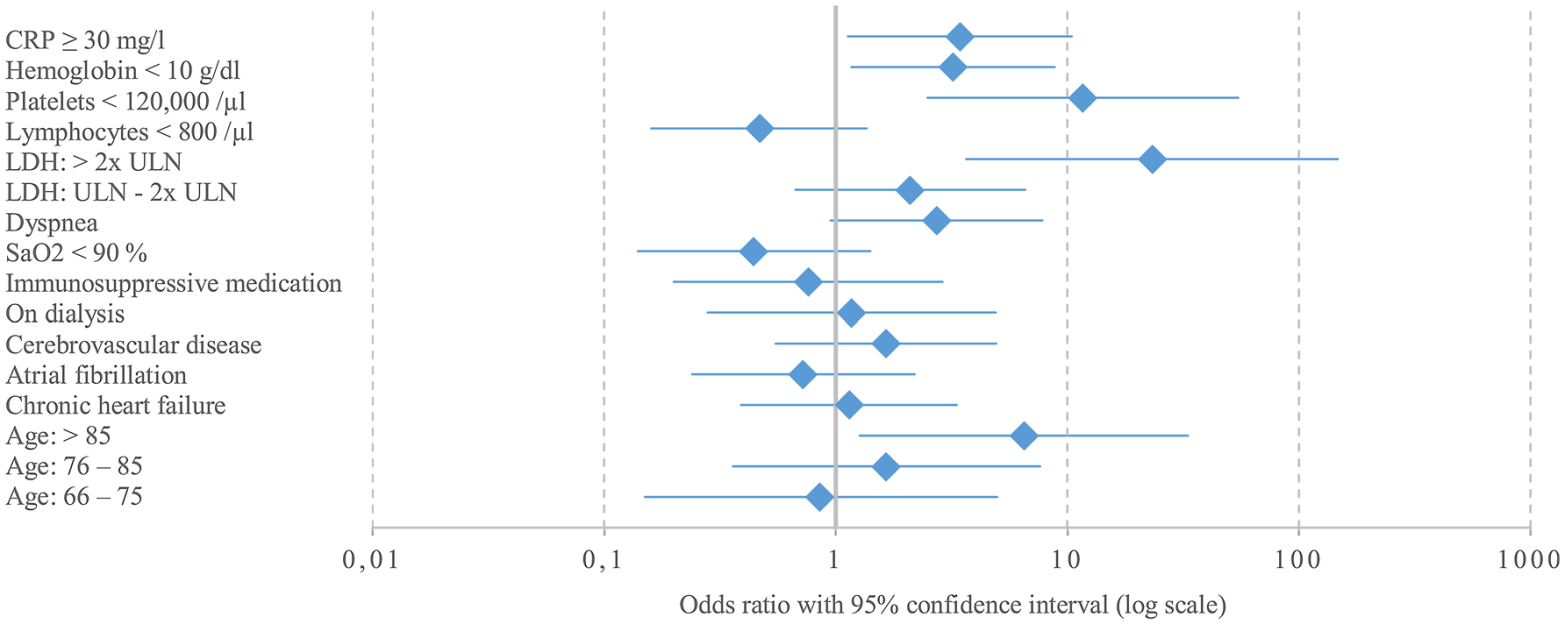
Forest plot of predictive factors for fatal outcome in SARS-CoV-2-infected patients suffering from chronic kidney disease. Continuous parameters were collected in categories. *n *= 289 observations were excluded from multivariable regression model due to missingness. Missing rates and frequency distribution are displayed in Suppl. Table 2 for variables with missing rate > 5%. Reference categories: CRP < 30 mg/l, hemoglobin ≥ 10 g/dl, platelets ≥ 120,000/µl, lymphocytes ≥ 800/µl, LDH normal, no dyspnea, SO2 ≥ 90%, no immunosuppressive medication, not on dialysis, no cerebrovascular disease, no atrial fibrillation, no chronic heart failure, age 15–65 years. *CRP* C-reactive protein, *SO*_*2*_ oxygen saturation in arterial blood, *LDH* lactate dehydrogenase, *ULN* upper limit of normal in the respective local laboratory

Our analyses included important baseline parameters; however, there might be further confounders which were not addressed in this study, and strong predictors which were not considered in the multivariable regression model due to their non-routine assessment in most settings (supplementary Table 4). Transferability to outpatient settings as well as to health care facilities beyond Germany may be limited as highest documentation was performed in German inpatient treatment settings (Fig. 2).

In conclusion, this comprehensive analysis of *LEOSS* registry identified characteristics of SARS-CoV-2-infected patients with CKD and predictive factors at initial presentation associated with unfavorable prognosis of COVID-19. The results obtained in this large multi-center cohort study indicate that mortality in CKD patients is independent of renal replacement therapy. Much more likely, the assessment of age, anemia, thrombocytopenia, LDH and CRP at first SARS-CoV-2 detection is crucial for predicting mortality in CKD patients, which may facilitate risk stratification for COVID-19 in high-risk CKD patients as early as at initial medical evaluation for SARS-CoV-2 and which are broadly available in both in- and outpatient settings throughout the world.

## Supplementary Information

Below is the link to the electronic supplementary material.

Supplementary file1 (DOCX 34 KB)

## Data Availability

The data the analyses of this study are based on can be requested from the corresponding author in justified cases. The access need to be discussed within and confirmed by the LEOSS Board of Investigators. A public dataset with a corresponding dashboard is available on the LEOSS homepage (https://leoss.net/data/).
