# Supplementary material for: Clinical course and predictive risk factors for fatal outcome of SARS-CoV-2 infection in patients with chronic kidney disease
Source: Infection. 2021 Apr 13;49(4):725–37. doi: 10.1007/s15010-021-01597-7 (PMC8043429; doi:10.1007/s15010-021-01597-7)
Supplement: Supplementary file 1 — Supplementary file1 (DOCX 34 KB) [file 15010_2021_1597_MOESM1_ESM.docx]

**Supplementary material - Infection**

**Clinical course and predictive risk factors for fatal outcome of SARS-CoV-2 infection in patients with chronic kidney disease**

Lisa Pilgram^1^*, Lukas Eberwein^2^*, Kai Wille^3^, Felix C. Koehler^4,5^, Melanie Stecher^6^, Siegbert R. Rieg^7^, Jan T. Kielstein^8^, Carolin E. M. Jakob^6^, Maria Ruethrich^9^, Volker Burst^4,10^, Fabian Prasser^11,12^, Stefan Borgmann^13^, Roman-Ulrich Müller^4,5,14^, Julia Lanznaster^15^, Nora Isberner^16^, Lukas Tometten^17+^, Sebastian Dolff^18+^

on behalf of the LEOSS Study group

^1^ Department of Internal Medicine, Hematology and Oncology, Goethe University Frankfurt, Frankfurt, Germany

^2^ 4th Department of Internal Medicine, Klinikum Leverkusen gGmbH, Leverkusen, Germany

^3^ University Clinic for Haematology, Oncology, Haemostaseology and Palliative Care, University of Bochum, Johannes Wesling Klinikum Minden, Germany

^4^ Department II of Internal Medicine and Center for Molecular Medicine Cologne, University of Cologne, Faculty of Medicine and University Hospital Cologne, Cologne, Germany

^5^ CECAD, University of Cologne, Faculty of Medicine and University Hospital Cologne, Cologne, Germany

^6^ University of Cologne, University Hospital of Cologne, Department I of Internal Medicine, Cologne, Germany

^7^ Department of Medicine II, University of Freiburg, Freiburg, Germany

^8^ Medical Clinic V, Academic Teaching Hospital Braunschweig, Braunschweig, Germany

^9^ Department of Internal Medicine II, University Hospital Jena, Jena, Germany

^10^ Emergency Department, Faculty of Medicine and University Hospital Cologne, University of Cologne, Cologne, Germany

^11^ Charite, University hospital Berlin

Berlin Institute of Health (BIH), Anna-Louisa-Karsch-Str. 2, 10178 Berlin, Germany

^12^ Charité – Universitätsmedizin Berlin, corporate member of Freie Universität Berlin, Humboldt-Universität zu Berlin, and Berlin Institute of Health

^13^ Department of Infectious Diseases and Infection Control, Ingolstadt Hospital, Ingolstadt, Germany

^14^ Systems Biology of Ageing Cologne (Sybacol), University of Cologne, Cologne, Germany

^15^ Department of Internal Medicine 2, Klinikum Passau, Passau, Germany

^16^ Division of Infectious Diseases, Department of Medicine II, University of Würzburg Medical Center, Würzburg, Germany

^17^ Department of Gastroenterology and Infectiology, Klinikum Ernst-von-Bergmann, Potsdam, Germany

^18^ Department of Infectious Diseases, University Hospital Essen, University Duisburg-Essen, Essen, Germany

* contributed equally

^+^ contributed equally

**Corresponding author:**

Sebastian Dolff, MD, PhD

Department of Infectious Diseases

University Hospital Essen

University Duisburg-Essen

Hufelandstr. 55

45122 Essen, Germany

Phone: +49-201-723-3394

Fax: +49-201-723-3395

[Sebastian.Dolff@uk-essen.de](mailto:Sebastian.Dolff@uk-essen.de)

Suppl. Table 1: Variance inflating factors of the covariables in the regression model in table 3.

| **Variables** | **VIF** |
| --- | --- |
| CRP ≥ 30 mg/l | 1.40 |
| Age: 76 - 85 | 1.90 |
| LDH: ULN - 2x ULN | 1.86 |
| Age: > 85 | 1.77 |
| Atrial fibrillation | 1.73 |
| Chronic heart failure | 1.62 |
| Age: 66 - 75 | 1.47 |
| Hemoglobin < 10 g/dl | 1.45 |
| LDH: > 2x ULN | 1.34 |
| Dyspnea | 1.34 |
| SO2 < 90 % | 1.32 |
| Cerebrovascular disease | 1.26 |
| Platelets < 120,000/µL | 1.20 |
| On dialysis | 1.19 |
| Immunosuppressive medication | 1.17 |

VIF, variance inflating factor. BMI, body mass index. CRP, C-reactive protein. LDH, lactate dehydrogenase. ULN, upper limit of normal in the respective local laboratory. SO2, oxygen saturation.

Suppl. Table 2: Missing rates and distribution among the outcome mortality.

|  | **Distribution of missing rate** | | | |
| --- | --- | --- | --- | --- |
|  | **Total** | **Mortality** | **Alive** | **p-value** |
| Chronic heart failure (%) | 6.3 | 5.0 | 7.0 | 0.960 |
| Coronary heart disease (%) | 6.8 | 7.9 | 6.3 | 0.985 |
| BMI (%) | 31.5 | 39.3 | 27.6 | 0.204 |
| Immunosuppressive drugs (%) | 12.0 | 14.3 | 10.8 | 0.901 |
| ACE inhibitors or ARBs (%) | 5.6 | 7.9 | 4.6 | 0.747 |
| Smoking status (%) | 51.9 | 50.0 | 52.8 | 0.990 |
| SO2 (%) | 24.9 | 22.1 | 26.2 | 0.933 |
| Temperature (%) | 22.8 | 20.7 | 23.8 | 0.973 |
| Hemoglobin (%) | 20.7 | 13.6 | 24.1 | 0.172 |
| Leukocytes (%) | 20.4 | 14.3 | 23.4 | 0.305 |
| Lymphocytes (%) | 38.3 | 37.1 | 38.8 | 0.999 |
| Platelets (%) | 21.4 | 15.0 | 24.5 | 0.285 |
| Creatinine (%) | 20.9 | 14.3 | 24.1 | 0.239 |
| Troponin T (%) | 68.5 | 75.0 | 65.4 | 0.402 |
| D dimer (%) | 70.9 | 73.6 | 69.6 | 0.948 |
| LDH (%) | 34.5 | 31.4 | 36.0 | 0.928 |
| CRP (%) | 21.4 | 15.7 | 24.1 | 0.412 |
| PCT (%) | 53.8 | 50.7 | 55.2 | 0.942 |
| Interleukin 6 (%) | 80.5 | 82.9 | 79.4 | 0.948 |
| Ferritin (%) | 72.8 | 83.6 | 67.5 | 0.370 |
| Urine leukocytes (%) | 62.9 | 62.9 | 62.9 | 1.000 |
| Urine hemoglobin (%) | 63.9 | 62.9 | 64.3 | 0.999 |
| Urine protein (%) | 64.8 | 66.4 | 64.0 | 0.993 |
| Dyspnea (%) | 20.9 | 20.7 | 21.0 | 1.000 |

BMI, body mass index. ACE inihibitors, angiotensin-converting enzyme inhibitor. ARBs, Angiotensin II receptor blocker. SO2, oxygen saturation in arterial blood. LDH, lactate dehydrogenase. CRP, C-reactive protein. PCT, procalcitonin.

Suppl. Table 3: Multivariable logistic regression of predictive factors for mortality in SARS-CoV-2 infected patients suffering from chronic kidney disease with strict selection of parameters within the univariate significance level of p < 0.05.

|  | **Mutivariable model** | | |
| --- | --- | --- | --- |
|  | **aOR** | **95% CI** | **p-value** |
| **Age - no. (%)** | | | |
| 15 – 65 | Reference | Reference | Reference |
| 66 – 75 | 0.82 | 0.21 - 3.25 | 0.775 |
| 76 – 85 | 1.51 | 0.46 - 4.92 | 0.495 |
| > 85 | 3.56 | 0.99 - 12.78 | 0.052 |
| **Sex - no. (%)** | | | |
| Female | * | * | * |
| Male | * | * | * |
| **BMI - no. (%)** | | | |
| < 18.5 | * | * | * |
| 18.5 – 24.9 | * | * | * |
| 25.0 – 29.9 | * | * | * |
| 30.0 – 34.9 | * | * | * |
| ≥ 35.0 | * | * | * |
| **Comorbidities - no. (%)^a^** | | | |
| Hypertension | * | * | * |
| Chronic heart failure | 1.58 | 0.69 - 3.66 | 0.283 |
| Atrial fibrillation | 0.81 | 0.34 - 1.96 | 0.641 |
| Coronary heart disease | * | * | * |
| Cerebrovascular disease | * | * | * |
| Diabetes mellitus | * | * | * |
| COPD | * | * | * |
| Oncological disease^b^ | * | * | * |
| **Dialysis - no. (%)^a^** |  |  |  |
| CKD on dialysis | * | * | * |
| **Smoking status - no. (%)** | | | |
| Active smoker | * | * | * |
| Former smoker | * | * | * |
| Non smoker | * | * | * |
| **Medication - no. (%)^a^** | | | |
| ACE inhibitors or ARBs^c^ | * | * | * |
| Immunosuppressive medication^d^ | * | * | * |
| **Vital signs^c^ - no. (%)^a^** | | | |
| Body temperature ≥ 38°C | * | * | * |
| SO2 < 90 % | * | * | * |
| Dyspnea | 2.09 | 0.91 - 4.83 | 0.083 |
| **LDH^c^ - no. (%)** | | | |
| Normal | Reference | Reference | Reference |
| ULN - 2x ULN | 1.41 | 0.61 - 3.28 | 0.423 |
| > 2x ULN | 4.90 | 1.28 - 18.72 | 0.020 |
| **Leukocytes^c^ - no. (%)** | | | |
| < 4,000 /µl | * | * | * |
| 4,000 - 11,999 /µl | * | * | * |
| > 12,000 /µl | * | * | * |
| **Lymphocytes^c^ - no. (%)^a^** | | | |
| < 800 /µl | 0.99 | 0.45 - 2.18 | 0.984 |
| **Platelets^c^ - no. (%)^a^** | | | |
| < 120,000 /µl | 7.77 | 2.39 - 25.26 | < 0.001 |
| **Hemoglobin^c^ - no. (%)^a^** | | | |
| < 10 g/dl | 3.22 | 1.43 - 7.24 | 0.005 |
| **CRP^c^ - no. (%)^a^** | | | |
| ≥ 30 mg/l | 2.09 | 0.93 - 4.70 | 0.074 |

Continuous parameters were collected in categories. All variables are expressed as numbers (no.) and percentages (%) referred to the numbers excluding missing data. Missing rates and frequency distribution are displayed in Suppl. table 2 for variables with missing rate > 5 %. n = 246 observations were excluded from multivariable regression model due to missingness. OR, odds ratio. aOR, adjusted odds ratio. CI, confidence interval. BMI, body mass index. COPD, chronic obstructive pulmonary disease. ACE inhibitors, angiotensin-converting enzyme inhibitor. ARBs, Angiotensin II receptor blocker. SO2, oxygen saturation in arterial blood. LDH, lactate dehydrogenase. ULN, upper limit of normal in the respective local laboratory. CRP, C-reactive protein.

* excluded due to missing significance in univariate modeling (p ≥ 0.05).

^a^ No reference level indicated in binary variables.

^b^ Leukemia, lymphoma or solid tumor.

^c^ At first positive SARS-CoV-2 detection.

^d^ Within the last 3 months.

Suppl. Table 4: Frequency distribution and univariate logistic regression of non-standard parameters in SARS-CoV-2 infected patients suffering from chronic kidney disease.

|  | **Frequency distribution** | | | **Univariate model** | | |
| --- | --- | --- | --- | --- | --- | --- |
|  | **Mortality** | **Alive** | **p-value** | **OR** | **95% CI** | **p-value** |
| **D dimer^a^ - no. (%)** | | | | | | |
| Normal | 3 (8.1) | 17 (19.5) | 0.310 | Reference | Reference | Reference |
| ULN - 2x ULN | 7 (18.9) | 29 (33.3) |  | 1.37 | 0.31 - 6.00 | 0.678 |
| > 2x ULN | 27 (73.0) | 41 (47.1) |  | 3.73 | 1.00 - 13.97 | 0.051 |
| **Inflammatory parameters^a^ - no. (%)^b^** | | | | | | |
| PCT > 0.5 ng/ml | 31 (44.9) | 28 (21.9) | 0.023 | 2.91 | 1.54 - 5.49 | < 0.001 |
| IL-6 ≥ 50 pg/ml | 20 (83.3) | 28 (47.5) | 0.061 | 5.54 | 1.69 - 18.18 | 0.005 |
| Ferritin ≥ 500 ng/ml | 15 (65.2) | 40 (43.0) | 0.456 | 2.48 | 0.95 - 6.43 | 0.061 |
| **Troponin T^a^ - no. (%)^c^** | | | | | | |
| Normal | 4 (13.3) | 37 (42.5) | 0.005 | Reference | Reference | Reference |
| ULN - 2x ULN | 5 (16.6) | 27 (31.0) |  | 1.71 | 0.42 - 6.98 | 0.453 |
| > 2x ULN | 21 (70.0) | 23 (26.4) |  | 8.45 | 2.57 - 27.74 | < 0.001 |

Continuous parameters were collected in categories. All variables are expressed as numbers (no.) and percentages (%) referred to the numbers excluding missing data. Missing rates and frequency distribution are displayed in Suppl. table 2 for variables with missing rate > 5%. ULN, upper limit of normal in the respective local laboratory. PCT, procalcitonin. IL6, interleukin 6.

^a^ At first positive SARS-CoV-2 detection.

^b^ No reference level indicated in binary variables.

^c^ Parameter only included in patients without dialysis due to unclear interpretation in dialysis patients.
